# Malarial Hæmorrhagic Fever

**Published:** 1884-12-20

**Authors:** J. K. Hodge

**Affiliations:** Princeton, Ark.


### MALARIAL HÆMORRHAGIC FEVER.

[Having been requested by a subscriber to copy an article on the above disease, from a western journal, we wrote to the author who kindly sent us a copy of the same. In his reply, he says :]

"My object in writing the article was to suppress the use of *quinine* in the treatment of this disease, which my observation had taught me was highly injurious, and whenever it is prescribed without the addition (accident in most cases) of some other agent which would counteract its deleterious effect upon the nervous system, death is almost invariably the result.

"Since writing the article in question, my observation has forced me to the conclusion that the disease, in most instances at least, originates from the abuse of quinine. In all of my experience, I have yet to see the first case of the disease in which quinine has not been freely used just previous to the development of the same.

"Some years ago, not willing to abandon so potent a remedy as quinine was then thought to be, but being somewhat suspicious as to its effects, I have suspended it in some cases, which would immediately begin to improve, and soon after convalescense was fully established, the resumption of the drug would bring about a return of all the aggravated symptoms."

By J. K. HODGE, M. D., of Princeton, Ark.

---

In January Brief, Dr. C. D. Arnold asked for most successful treatment for malarial hæmorrhagic fever of the Southern States, which was very promptly responded to by Dr. Sikes, in March number of same, page 109, with whom I beg to take issue through the columns of your journal.

The doctor would have to begin treatment with large doses of quinine, repeated at short intervals, at the same time giving 10-drop doses of dil. mur. acid, which last I consider of no minor importance in his plan of treatment, since it is called upon to exert an influence in counteracting the poisonous effects of a poison which he would persist in administering in 5-grain doses every hour.

In this brief article I propose giving what I consider the most successful treatment of said disease, and that, too, without a single grain of quinine or any of its salts, either at the beginning or end of treatment. I say this is the most successful, from the fact that, in the fall of 1880, my colleague, Dr. Parham, and myself were called upon to treat more than forty cases of the disease, some of which were in its most malignant form—all of which we treated successfully, with one single exception, and this case having some aggravated complications. At the same time, and under the same surrounding influence, our brother practitioners, who would persist in the quinine treatment, lost from 25 to 50 per cent. of their patients!

This evidence was too preponderant not to be heeded; consequently, the profession generally, in our section, have adopted the same plan of treatment, and now no longer look upon the disease with any degree of alarm. Since the date above mentioned I have not been called to treat so many cases in same length of time, but treat a number of cases every season, with proportionate success.

Our plan of treatment is to apply, as early as possible, a large blister over the epigastrium, after which I prescribe as follows:

℞ Hydrarg. Chlor. Mit........................ 6 grains
Pulv. Doveri..............................30 "
Plumbi. Acetas............................ 3 "

M. et div. six powders.

Sig.: One powder to be placed on the tongue every four hours, until the whole are taken.

This I alternate with fl. ext. ergot, in 30-drop doses, to be given every four hours also. Should the ergot not be tolerated by the stomach—which is sometimes the case—I substitute therefor tr. mur. iron, in 30-drop doses, which acts with almost equal efficiency.

By the time this prescription is exhausted the vomiting, hemorrhage, and all other aggravated symptoms have, generally, subsided, and I proceed to place the patient on tonic treatment, as follows:

℞. Tr. Ferri. Mur............................ 1 ounce
Dil. Phos. Acid.......................... 7 drachms
Tr. Nucis. Vom......................... 1 drachm.

M. Sig.: Thirty drops every four hours for forty-eight hours; after which, three times a day.

Now, brethren, you see that I do not prescribe quinine at any stage of the disease, and I am zealous in contending that, until this plan of treatment be adopted generally by the profession, the disease will continue to be a terror to the land. I fain would lengthen this article to twice its proportions, but am admonished that I must be brief.

